# Supplementary material for: Effect of Chemical Treatment of Cotton Stalk Fibers on the Mechanical and Thermal Properties of PLA/PP Blended Composites
Source: Polymers (Basel). 2024 Jun 10;16(12):1641. doi: 10.3390/polym16121641 (PMC11207778; doi:10.3390/polym16121641)
Supplement: Supplementary file 1 [file polymers-16-01641-s001.zip › polymers-3007943-supplementary.pdf]

polymers

MDPI

Supplementary Information

Effect of Chemical Treatment of Cotton Stalk Fibers on the Mechanical and Thermal Properties of PLA/PP Blended Composites

Feng Xu [1,†], Jin Shang [1,†], Abdukeyum Abdurexit [2], Ruxangul Jamal [2,*], Tursun Abdiryim [1,*], Zhiwei Li [2], Jiangan You [1], Jin Wei [1], Erman Su [2] and Longjiang Huang [2]

1 State Key Laboratory of Chemistry and Utilization of Carbon Based Energy Resources, College of Chemistry, Xinjiang University, Urumqi 830017, China; feng.xu@xju.edu.cn (F.X.); shangjinpop@163.com (J.S.); jayou@xju.edu.cn (J.Y.); 17793738494@163.com (J.W.)
2 State Key Laboratory of Chemistry and Utilization of Carbon Based Energy Resources, State Key Laboratory of Oil and Gas Fine Chemicals, Ministry of Education & Xinjiang Uygur Autonomous Region, Xinjiang University, Urumqi 830017, China; abdukaiyum@sohu.com (A.A.); li2812355161@163.com (Z.L.); suerman47@163.com (E.S.); 18034152696@163.com (L.H.)
* Correspondence: jruxangul@xju.edu.cn (R.J.); tursunabdir@xju.edu.cn (T.A.)
† These authors contributed equally to this work.

The detailed thermal and crystallinity data were obtained through the analysis of differential scanning calorimetry, as presented in Table S1~S3.

Table.S1 Thermal properties for composites obtained from DSC first heating run experiments.

Sample	T_g (°C)	T_c (°C)	ΔH_c(J/g)	T_m (°C)	ΔH_m (J/g)	X_c (%)
UN-CSF	55.03	86.85	18.93	166.13	43.21	32.39
SA-CSF	56.98	85.47	19.84	165.14	49.22	39.19
N-CSF	56.49	88.14	17.69	165.15	47.23	39.40
S-CSF	55.67	86.36	20.98	166.81	47.82	35.81
NS-CSF	57.93	87.29	11.76	165.41	45.40	44.87

Table.S2 Thermal properties for composites obtained from DSC second heating run experiments.

Sample	T_g (°C)	T_c (°C)	ΔH_c(J/g)	T_m (°C)	ΔH_m (J/g)	X_c (%)
UN-CSF	59.47	92.82	16.07	166.27	43.20	36.19
SA-CSF	58.52	91.74	21.25	165.66	48.54	36.40
N-CSF	58.95	92.51	24.86	165.29	45.78	27.91
S-CSF	58.94	92.70	20.25	166.68	48.16	37.23
NS-CSF	58.98	92.55	18.40	165.75	43.79	33.87

Table.S3 Thermal properties for composites obtained from DSC cooling run experiments.

Sample	T_{c1} (°C)	ΔH_{c1}(J/g)	T_{c2} (°C)	ΔH_{c2}(J/g)
UN-CSF	88.82	13.57	122.80	5.11
SA-CSF	88.42	13.58	122.73	4.94
N-CSF	-	-	123.17	4.82
S-CSF	89.84	14.91	122.45	4.86
NS-CSF	88.23	13.19	124.02	4.71
